# Seasonal Variation: A Non-negligible Factor Associated With Blood Pressure in Patients Undergoing Hemodialysis

**DOI:** 10.3389/fcvm.2022.820483

**Published:** 2022-03-18

**Authors:** Zhibin Wu, Shan Lan, Chengqiang Chen, Xiuan Zhang, Yazhen Zhang, Shanying Chen

**Affiliations:** ^1^Department of Nephrology, Zhangzhou Affiliated Hospital of Fujian Medical University, Zhangzhou, China; ^2^Hemodialysis Unit, Zhangzhou Affiliated Hospital of Fujian Medical University, Zhangzhou, China

**Keywords:** seasonal variation, blood pressure, hemodialysis, hypertension, pre-dialysis, DN

## Abstract

**Objective:**

To investigate a seasonal variation in blood pressure (BP) for patients undergoing hemodialysis (HD).

**Methods:**

In this retrospective study, we exported all BP measurements from the information system to investigate a seasonal variation of BP. We also investigated a seasonal variation in BP for patients of different gender types, of different age groups, with diabetic nephropathy (DN), and with non-DN having HD. Multiple linear regression models were used to explore the associations between BP and climatic parameters.

**Results:**

In 2019, a total of 367 patients had received HD therapy in the Longwen HD unit. We included nearly 40,000 pre-dialysis BP measurements. The result of our study demonstrated a clear seasonal variation in pre-dialysis BP in general patients with HD, in male and female patients, and patients with DN and non-DN. December seemed to be a peak in the values of pre-dialysis systolic BP (SBP) and diastolic BP (DBP). The nadir values of pre-dialysis SBP and DBP were observed in June and July, respectively. A difference between peak and nadir values of BP is 3.81/2.20 mmHg in patients undergoing HD. Maximal seasonal variation in BP is 9.03/5.08 mmHg for patients with DN. A significant association of SBP and DBP with climatic parameters was found in this study. Pre-dialysis BP was inversely correlated with outdoor temperature, daytime length, and relative humidity.

**Conclusion:**

A clear seasonal variation in BP is observed for patients with HD. Pre-dialysis SBP and DBP are inversely associated with outdoor temperature, daytime length, and relative humidity. The magnitude of a seasonal variation in BP increases in patients with DN.

## Introduction

Although renal replacement therapy can effectively prolong the survival rate of patients with end-stage kidney disease (ESRD), the 5-year mortality of the maintenance of patients with hemodialysis (HD) is still reached up to 32.3–25.9% (1). Blood pressure (BP) status is considered a key mediator of outcomes in patients with dialysis (2). Cardiovascular disease is the leading cause of death in patients with dialysis (2, 3). Uncontrolled hypertension contributes to an increase in the mortality rate of patients with dialysis (2, 3). The pathogenesis of hypertension in patients with dialysis is complex and multifactorial (2, 4, 5). It was first documented that a seasonal variation in outdoor temperature was associated with BP in 1961 (6). Thereafter, a considerable number of studies indicated the impact of a seasonal variation on BP in the general population, in the male and female population, and all age group individuals (7). Nevertheless, a seasonal BP variation is often neglected in clinical practice and clinical studies (7, 8). In 1998, Argilés reported that BP varies seasonally, with higher values in the winter and lower values in the summer in patients with HD (9). Fourteen studies investigated a seasonal BP variation in patients with HD, which were summarized in a previous consensus document (7). With the exception of Adrian Fine's study (10), other studies reported a substantial seasonal systolic BP (SBP) variation in patients with HD (7). In Adrian Fine's study, the result indicated that seasonal changes in BP may be nonclimatic-related (10). A possible explanation for such a divergence included different climate types, older adult patients, and different prevalences of hypertension (10). Data from the Dialysis Outcomes and Practice Patterns Study (DOPPS) on years 2005–2011 indicated that not only a seasonal variation but also a geographical gradient influence the BP in European patients with HD (11). A seasonal variation in BP may also be affected by different geographical gradients and climate types (11). Both seasonal variation and geographical gradient are nonnegligible factors for BP control. As we know, a seasonal BP variation in patients with HD has not been investigated in the Chinese population. In consideration of numerous studies on hypertension in patients with HD, seasonal factors are obviously neglected in clinical practice. Our study attempts to fill the gap in this dilemma. In the present retrospective study, we included a great quantity of data on BP in our HD unit. The aim of the present study is to investigate a seasonal variation in BP for patients who are undergoing HD. We also investigate a seasonal variation of BP in different gender types, different age groups, diabetic nephropathy (DN), and non-DN patients with HD. Data on BP included pre-dialysis BP, post-dialysis BP, and intradialytic BP.

## Methods

### Ethics Statement

The present study has been approved by the Ethics Committee of Zhangzhou Affiliated Hospital of Fujian Medical University (2021LWB165). The declaration of Helsinki of the World Medical Association was followed in this study.

### Data Collection

Our study period spanned from January 1, 2019, to December 31, 2019. In our previous study, we have described our dialysis center and therapy method (12). Different from our previous study, we only used the data on one of our HD units (Longwen HD unit) in this study. We directly output the data on BP measurements, ultrafiltration volume (UV), and other relevant information from our information system. We used the outpatient information system to collect the use of antihypertensive drugs. These drugs included calcium antagonists (CCBs), angiotensin II receptor blockers (ARB), angiotensin-converting enzyme inhibitors (ACEI), β-blockers, clonidine, and an α-receptor antagonist.

### Data on BP Measurements

During HD therapy, the patients received BP measurements at least five times, including pre-dialysis BP, post-dialysis BP, and intradialytic BP measurements at least once an hour. The dialysis machine (Dialog+710200R & Dialog+710207T, B. Braun, Germany) has a device, which was used for automatically measuring BP. The device is an electronic sphygmomanometer module composed of an inflation pump, a solenoid valve, a pipeline, a filter screen, a circuit IC, and a display module. The method of BP measurement was presented as follows: (1) the BP cuff was wrapped around the patients' upper arm; (2) automatically pressurize the cuff to block the brachial artery blood; and (3) slowly reduce the pressure. During BP measurement, the arm sent out sound and pressure, which were recognized by an HD machine. The receiver converted them into electrical signals. Finally, it was converted into digital signals (BP values). When BP was measured, patients were in the supine position. BP measurements were automatically output and stored in the information system.

We exported all BP measurements from the information system. Inclusion criteria included (1) patients diagnosed as ESRD by nephrologists and (2) patients receiving HD in the Longwen HD unit from January 1, 2019, to December 31, 2019. We excluded BP measurements when patients initiated dialysis therapy <90 days. We also excluded BP measurements if pre-dialysis SBP was <60 mmHg or diastolic BP (DBP) was <30 mmHg because these were probably the measurement errors.

### Definition

Target values of pre-dialysis and post-dialysis BP in patients with HD were ≤ 140/90 and ≤ 130/80 mmHg, respectively (13). Intradialytic hypertension was defined as an SBP increase of >10 mmHg from pre-dialysis to post-dialysis (14).

### Climatic Parameters

Climatic parameters were obtained from the Zhangzhou City Weather Website^1^,^2^ and a meteorological website^3^. Daily maximal temperature, minimum temperature, and daytime length were obtained from the Zhangzhou City Weather Website^1^,^2^. We used month as a unit and monthly calculated the means of daily maximal temperature, daily minimum temperature, and day length. The means of monthly outdoor temperature and relative humidity were obtained from the meteorological website^3^. We used the geographical location of Zhangzhou (117.65° east longitude and 24.55° north latitude) to query information.

During HD therapy, the indoor temperature was adjusted by the air conditioners in the winter and summer. The temperature of the air conditioner in the winter was set as 25°C and 20°C in the summer, maintaining the indoor temperature of about 25°C. The routine setting temperature of an HD machine was 36–37°C.

### Other Data

Data on age, gender, primary diseases, HD vintage, vascular access, and other clinical variables were also collected. UV per HD was collected from the information system.

### Data Analysis

Blood pressure measurements, UV, and pulse were derived from the information system. We used Excel software to collect and organize data. *Stata* statistical software (version 12.0) and Excel software were used to perform data analysis and plot figures. We calculated the means of monthly pre-dialytic and post-dialytic SBP and DBP. The variables were presented as means ± SD. The coefficient of variation (CV) was also calculated. The prevalence of intradialytic hypertension was calculated on a monthly basis. Means of daily maximum temperature, daily minimum temperature, daily temperature, daily daytime length, and relative humidity were also calculated on a monthly basis. One-way ANOVA was used to compare differences in BP (pre-dialysis, post-dialysis SBP, and DBP) for each month.

All of the data on BP measurements were grouped by gender, different age groups, and DN/non-DN subgroups. The abovementioned statistical analysis was repeated in men and women, different age groups, and DN and non-DN subgroups. A paired *t*-test was used to compare pre-dialysis and post-dialysis BP. The student's *t*-test was used to compare pre-dialysis BP between the different subgroups (male and female; DN and non-DN).

Multiple linear regression models were used to explore the associations between BP measurements and climatic parameters. In the models, BP measurements (pre-dialysis SBP or DBP) were used as independent variables and climatic parameters were used as dependent variables. The models were adjusted for age, gender, primary diseases, vascular access, HD vintage, and UV. The value of *p* < 0.05 was considered statistically significant.

## Results

In 2019, a total of 367 patients had received HD therapy in the Longwen HD unit. By December 31, 2019, 356 patients had received HD ≥ 90 days. The mean age of the 356 patients was 53 years. The ratio of male/female was 202/154. By December 31, 2019, the median HD vintage was 29 months (mean HD vintage: 39 months). Major primary diseases were glomerulonephritis (109/356) and DN (87/356). In total, 330 patients used autologous arteriovenous fistula as vascular access.

In 2019, 41,278 HD therapies were completed in the Longwen HD unit. We excluded 14 BP measurements because pre-dialysis SBP was <60 mmHg or DBP was <30 mmHg. We excluded HD therapies when the patients initiated the dialysis <90 days. Finally, a total of 38,970 cases of HD therapies were included in this study. In 2018–2019, 208 patients received echocardiography examinations. A total of 21 (10.10%) had a reduced ejection fraction (<50%). Nine patients with a reduced ejection fraction had hypertension. The mean pre-dialysis pulse was 80.08 ± 14.53 /min.

### Pre-dialysis and Post-dialysis BP

Mean pre-dialysis and post-dialysis BP measurements were calculated on a monthly basis. The results were presented in Table 1. Mean pre-dialysis and post-dialysis BPs were 146.07/85.82 and 142.85/85.79 mmHg, respectively. Pre-dialysis SBP was significantly higher than post-dialysis SBP (*p* < 0.05). However, we did not find a significant difference between pre-dialysis and post-dialysis DBP.

**Table 1:** Pre-dialysis blood pressure (BP), post-dialysis BP, intradialytic hypertension, and hemodialysis (HD) ultrafiltration volume (UV) in patients receiving HD.

**Month**	***N***	**Pre-dialysis SBP (mmHg)**	**Pre-dialysis DBP (mmHg)**	**Post-dialysis SBP (mmHg)**	**Post-dialysis DBP (mmHg)**	**Intradialytic hypertension (%)**	**Ultrafiltration volume (ml)**
Jan.	3,090	146.79 ± 23.31	85.58 ±16.82	142.99 ± 22.61	84.92 ± 15.48	22.59 (21.11, 24.06)	2941 ± 1034
Feb.	2,787	146.66 ± 23.53	85.34 ±16.77	142.80 ± 23.81	85.17 ± 16.19	21.89 (20.35, 23.42)	3,010 ± 1,028
Mar.	3,075	147.36 ± 22.47	86.43 ± 16.85	143.46 ± 23.01	85.85 ± 16.18	20.88 (19.44, 22.32)	2,957 ± 1,013
Apr.	3,131	145.44 ± 22.73	85.13 ± 16.37	143.90 ± 23.42	85.99 ± 15.74	25.93 (24.40,27.47)	2,872 ± 1,042
May	3,305	145.29 ± 21.71	85.66 ± 16.51	143.88 ± 22.63	86.32 ± 15.32	25.63 (24.14, 27.12)	2,838 ± 1,012
Jun.	3,179	144.48 ± 22.45	85.20 ± 16.25	144.38 ± 23.50	86.80 ± 15.91	27.05 (25.51, 28.60)	2,749 ± 1,006
Jul.	3,492	144.65 ± 23.13	84.79 ±16.12	144.57 ± 23.92	86.34 ± 15.71	28.09 (26.60, 29.58)	2,739 ± 986
Aug.	3,423	144.91 ± 23.41	85.44 ±16.32	143.47 ±23.96	86.53 ± 16.02	25.09 (23.64, 26.55)	2,781 ± 945
Sept.	3,127	145.27 ± 23.04	86.00 ± 16.89	142.42 ± 23.52	86.22 ± 15.95	23.41 (21.92, 24.89)	2,837 ± 975
Oct.	3,484	146.41 ± 23.23	86.56 ±16.84	141.54 ± 23.97	85.74 ± 5.90	20.92 (19.57, 22.28)	2,941 ± 989
Nov.	3,426	147.28 ± 23.66	86.54 ± 16.03	140.75 ± 23.50	84.89 ±15.78	19.06 (17.74, 20.38)	2,948 ± 957
Dec.	3,451	148.29± 23.27	86.99 ± 16.48	140.34 ± 23.24	84.71 ±15.61	17.53 (16.26, 18.80)	2,998 ± 956
Total	3,8970	146.07 ± 23.03	85.82 ±16.53	142.85 ± 23.47	85.79 ± 15.82	23.16 (22.75, 23.59)	2,880 ± 998

Among 38,970 pre-dialysis BP measurements, 22,627 (58.06%) pre-dialysis SBP measurements were higher than 140 mmHg. A total of 13,827 (35.48%) pre-dialysis DBP measurements were higher than 90 mmHg. Totally, 27,574 (70.76%) post-dialysis SBP measurements and 23,914 (61.37%) post-dialysis DBP measurements were >130 and >80 mmHg, respectively. In 38,970 HD therapies, the prevalence of intradialytic hypertension was 23.17% (9,028). Pre-dialysis and post-dialysis BP measurements in men and women, different age groups, and DN/non-DN subgroups were also presented in Tables 2–4. CVs of pre-dialysis SBP and DBP were 15.77% and 19.26%, respectively (Supplementary Material 1).

**Table 2:** Pre-dialysis BP, post-dialysis BP, intradialytic hypertension, and HD UV in men and women.

	***N***	**Pre-dialysis SBP (mmHg)**	**Pre-dialysis DBP (mmHg)**	**Post-dialysis SBP (mmHg)**	**Post-dialysis DBP (mmHg)**	**Intradialytic hypertension (%)**	**Ultrafiltration volume (ml)**
**Male**	22,211	146.51 ± 22.79	86.47 ± 15.95	143.13 ± 23.26	86.53 ± 15.35	22.38 (21.83, 22.93)	3,069 ± 1,024
Jan.	1,737	147.63 ± 22.68	86.62 ± 16.22	143.70 ± 22.43	85.86 ± 15.37	21.42 (19.49, 23.35)	3,158 ± 1,041
Feb.	1,588	147.16 ± 23.31	86.12± 16.31	142.72 ± 23.12	85.93 ± 15.71	20.34 (18.36, 22.32)	3,222 ± 1,028
Mar.	1,765	147.78 ± 21.77	87.01 ± 15.76	143.85 ± 22.56	86.56 ± 15.52	20.23 (18.35, 22.10)	3,166 ± 1,041
Apr.	1,773	146.49 ± 22.14	86.15 ± 15.21	145.23 ± 22.64	87.41± 14.94	25.76 (23.74, 27.81)	3,071 ± 1,070
May	1,899	146.50 ± 20.79	87.12± 15.46	145.44 ± 21.38	87.93 ± 14.48	24.91 (22.96, 26.85)	3,063 ± 1,037
Jun.	1,823	145.25± 21.91	86.39 ± 15.83	145.35 ± 22.63	88.10 ± 15.20	27.43 (25.38, 29.48)	2,945 ± 1,008
Jul.	1,962	144.93± 23.17	85.37± 15.72	144.88 ± 24.05	87.28 ± 15.14	27.78 (25.69, 29.76)	2,925 ± 979
Aug.	1,942	144.87 ± 23.41	85.58 ± 15.61	143.15± 24.36	86.80± 15.43	23.33 (21.44, 25.21)	2,956 ± 967
Sept.	1,789	145.77 ± 23.47	86.67 ± 16.24	142.67 ± 24.10	87.02 ± 15.63	22.14 (20.21, 24.06)	2,988 ± 1,032
Oct.	1,973	146.48 ± 23.44	86.64 ± 16.49	140.68 ± 23.81	85.63± 15.13	19.51 (17.76, 21.26)	3,123 ± 1,040
Nov.	1,972	147.29± 23.77	86.61 ± 16.29	140.49 ± 23.31	85.08 ± 15.46	19.17 (17.43, 20.91)	3,111 ± 997
Dec.	1,988	148.13 ± 23.08	87.13 ± 16.18	139.92 ± 23.44	84.89 ± 15.79	16.70 (15.06, 18.34)	3,157 ± 1,014
Female	1,6759	145.48± 23.33	84.95± 17.22	142.48 ± 23.75	84.82 ± 16.38	24.21 (23.56, 24.86)	2,637 ± 907
Jan.	1,353	145.71 ± 24.06	84.24± 17.47	142.07 ± 22.80	83.72 ± 15.55	24.09 (21.81, 26.38)	2,685 ± 967
Feb.	1,199	146.00 ± 23.81	84.30± 17.30	142.90 ± 24.71	84.16 ± 16.76	23.94 (21.52, 26.36)	2,734 ± 962
Mar.	1,310	146.79 ± 23.38	85.64± 18.20	142.92 ± 23.60	84.89 ± 16.99	21.76 (19.52, 23.99)	2,689 ± 910
Apr.	1,358	144.08 ± 23.40	83.80± 17.70	142.18 ± 24.30	84.14 ± 16.56	26.14 (23.80, 28.48)	2,629 ± 952
May	1,406	143.66± 22.81	83.69± 17.65	141.78 ± 24.07	84.16 ± 16.13	26.60 (24.29, 28.91)	2,547 ± 900
Jun.	1,356	143.44 ± 23.12	83.60 ± 16.67	143.07 ± 24.57	85.05 ± 16.67	26.55 (24.20, 28.90)	2,497 ± 946
Jul.	1,530	144.29 ± 23.08	84.05 ± 16.58	144.17 ± 23.75	85.14± 16.33	28.50 (26.23, 30.76)	2,505 ± 944
Aug.	1,481	144.94 ± 23.41	85.26 ± 17.22	143.90 ± 23.42	86.18± 16.76	27.42 (25.14, 29.69)	2,549 ± 862
Sept.	1,338	144.61 ± 22.44	85.10 ± 17.68	142.09 ± 22.73	85.15 ± 16.32	25.11 (22.79, 27.44)	2,632 ± 852
Oct.	1,511	146.32 ± 22.96	86.44 ± 17.30	142.66 ± 24.15	85.88± 16.78	22.77 (20.65, 24.88)	2,706 ± 865
Nov.	1,454	147.26 ± 23.51	86.17 ± 15.67	141.09 ± 23.76	84.64± 16.19	18.91 (16.90, 20.93)	2,733 ± 856
Dec.	1,463	148.51 ± 23.54	86.80 ± 16.88	140.91 ± 22.96	84.45 ± 15.47	18.66 (16.66, 20.66)	2,776 ± 820

**Table 3:** Pre-dialysis BP, post-dialysis BP, intradialytic hypertension, and HD UV in patients with DN and non-DN.

	***N***	**Pre-dialysis SBP (mmHg)**	**Pre-dialysis DBP (mmHg)**	**Post-dialysis SBP (mmHg)**	**Post-dialysis DBP (mmHg)**	**Intradialytic hypertension (%)**	**Ultrafiltration volume (ml)**
DN	9,704	155.24 ± 22.98	83.06± 16.23	148.89± 23.03	81.86 ± 15.23	22.62 (21.79, 23.45)	3,156 ± 974
Jan.	747	155.31 ± 23.10	83.72± 16.90	150.76 ± 22.74	81.45 ± 14.12	26.24 (23.08, 29.40)	3,226 ± 1,065
Feb.	680	155.97 ± 24.76	83.14± 16.94	150.58 ± 25.29	82.83 ± 17.11	22.06 (18.93, 25.18)	3,332 ± 1,069
Mar.	704	156.67 ± 23.17	83.77± 17.20	150.15 ± 23.57	81.78 ± 16.01	20.60 (17.60, 23.59)	3,289 ± 998
Apr.	734	155.18 ± 23.20	82.03± 15.49	150.43 ± 23.63	81.72 ± 16.03	24.52 (21.40, 27.64)	3,222 ± 1,009
May	825	153.80 ± 20.26	82.12 ± 16.40	149.28 ± 22.37	81.60 ± 15.16	24.85 (21.89, 27.80)	3,119 ± 974
Jun.	792	151.21 ± 21.87	80.36± 14.15	148.86 ± 22.43	81.10 ± 15.35	28.41 (25.26, 31.56)	3,020 ± 999
Jul.	887	153.21± 22.90	81.26 ± 14.96	150.30 ± 22.63	82.42 ± 15.33	27.28 (24.35, 30.22)	3,053 ± 1,001
Aug.	889	155.08 ± 23.21	82.80 ± 16.59	150.43 ± 23.31	83.10± 15.74	24.41 (21.58, 27.24)	3,056 ± 926
Sept.	816	153.22 ± 22.41	82.63 ± 16.59	147.22 ± 22.10	81.93 ± 14.87	22.18 (19.32, 25.04)	3,094 ± 929
Oct.	896	155.16 ± 22.34	84.10 ±15.62	147.00 ± 22.53	81.95 ± 14.17	19.42 (16.82, 22.01)	3,171 ± 932
Nov.	876	157.80 ± 23.93	85.13 ± 16.76	146.46 ± 22.70	81.53 ± 14.91	17.35 (14.84, 19.86)	3,163 ± 887
Dec.	858	160.24 ± 23.38	85.44 ± 16.94	146.00 ± 22.77	80.91± 14.20	14.92 (12.53, 17.31)	3,220 ± 901
Non-DN	29,266	143.02 ± 22.23	86.73± 16.52	140.85 ± 23.27	87.10 ± 15.80	23.34 (22.86, 23.83)	2,789 ± 989
Jan.	2,343	144.08 ± 22.72	86.17 ± 16.76	140.51 ± 22.00	86.03 ± 15.73	21.43 (19.76, 23.09)	2,853 ± 1,009
Feb.	2,107	143.66 ± 22.31	86.05 ± 16.65	140.29± 22.76	85.92 ± 15.81	21.83 (20.07, 23.60)	2,906 ± 993
Mar.	2,371	144.59 ± 21.50	87.21 ± 16.67	141.47 ± 22.47	87.05 ± 16.03	20.96 (19.32, 22.60)	2,858 ± 997
Apr.	2,397	142.46 ± 21.73	86.08 ± 16.52	141.81 ± 22.96	87.30 ± 15.42	26.37 (24.60, 28.13)	2,768 ± 1,029
May	2,480	142.46 ± 21.44	86.84 ± 16.39	142.09 ± 22.43	87.90± 15.05	25.89 (24.16, 27.61)	2,748 ± 1,008
Jun.	2,387	142.25 ± 22.19	86.81 ± 16.59	142.89 ± 23.66	88.69 ± 15.64	26.60 (24.83, 28.38)	2,658 ± 992
Jul.	2,605	141.74 ± 22.48	85.99 ± 16.32	142.62 ± 24.04	87.68 ±15.62	28.37 (26.64, 30.10)	2,634 ± 958
Aug.	2,534	141.34 ± 22.41	86.36 ± 16.31	141.03 ± 23.71	87.73± 15.94	25.34 (23.64, 27.03)	2,685 ± 933
Sept.	2,311	142.47 ± 22.60	87.18± 16.83	140.72 ± 23.77	87.73 ± 16.05	23.84 (22.10, 25.58)	2,744± 975
Oct.	2,588	143.38 ± 22.76	87.41 ± 17.17	139.65 ± 24.17	87.06± 16.20	21.45 (19.86, 23.03)	2,858 ± 996
Nov.	2,550	143.67 ± 22.46	87.02 ± 15.75	138.78 ± 23.46	86.04 ± 15.90	19.65 (18.10, 21.19)	2,871 ± 969
Dec.	2,593	144.34 ± 21.85	87.51 ± 16.30	138.46 ± 23.09	85.96± 15.85	18.40 (16.90, 19.89)	2,920 ± 963

**Table 4:** Pre-dialysis BP, post-dialysis BP, intradialytic hypertension, and HD UV according to different age groups.

**Age (years)**	**N**	**Pre-dialysis SBP (mmHg)**	**Pre-dialysis DBP (mmHg)**	**Post-dialysis SBP (mmHg)**	**Post-dialysis DBP (mmHg)**	**Intradialytic hypertension (%)**	**Ultrafiltration volume (ml)**
<20	328	134.58 ± 15.17	88.01 ± 12.05	139.93 ± 17.03	92.92± 11.82	25.61 (20.86, 30.36)	2,121 ± 807
20–29	969	139.10± 18.63	85.95 ± 15.22	134.50 ± 20.80	86.22 ± 16.11	14.55 (12.33, 16.78)	2,815 ± 987
30–39	7,522	144.00 ± 22.08	91.38 ± 17.02	138.77 ± 23.66	89.88 ± 17.13	16.91 (16.06, 17.76)	3,105 ± 852
40–49	7,587	144.44 ± 22.63	87.91± 15.27	142.22 ± 24.19	87.66 ± 15.22	23.12 (22.17, 24.07)	3,176 ± 1,080
50–59	11,218	150.25 ± 21.69	87.44 ± 15.14	145.64 ± 22.19	86.97± 14.23	22.27 (21.50, 23.04)	2,946 ± 1,038
60–69	7,223	146.65 ± 24.80	79.25 ± 14.86	142.57 ± 23.14	79.77± 14.11	24.59 (23.59, 25.58)	2,681 ± 889
≥70	4,123	142.96 ± 25.12	78.67± 18.66	146.55 ± 24.99	81.60 ± 17.51	36.45 (34.98, 37.92)	2,256 ± 764

### The Use of Antihypertensive Drugs

The use of antihypertensive drugs was listed on a monthly basis (Supplementary Material 2). In 2019, 132 patients experienced a decrease in doses of antihypertensive drugs, and 88 patients experienced an increase in doses of antihypertensive drugs. The number of patients for which antihypertensive drug doses began to decrease or increase was presented in Supplementary Material 3. We did not find significant differences in antihypertensive doses among 12 months. However, compared with June, the doses of CCB and clonidine were higher in December.

### Seasonal Variations in Pre-dialysis SBP and DBP

**Figure 1:**
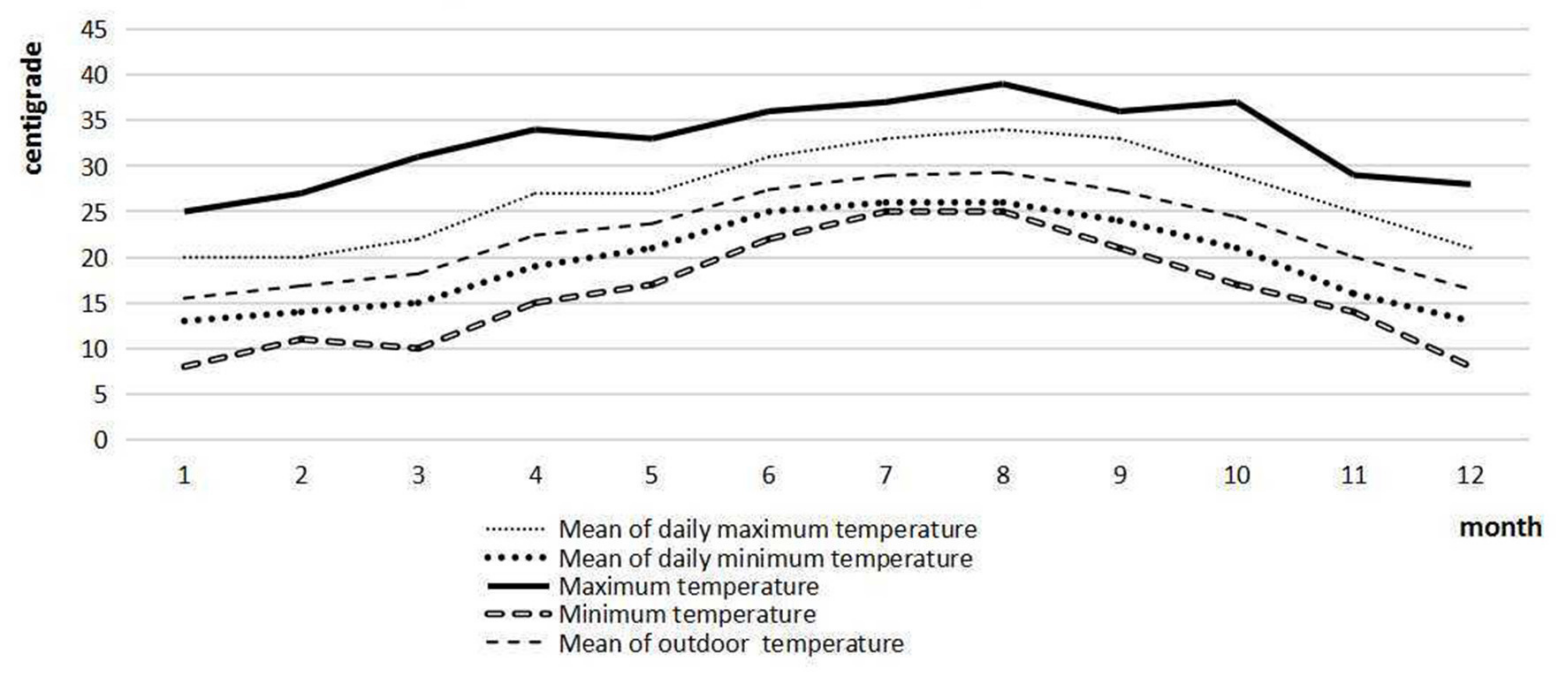
Temperature parameters of Zhangzhou in 2019.

**Figure 2:**
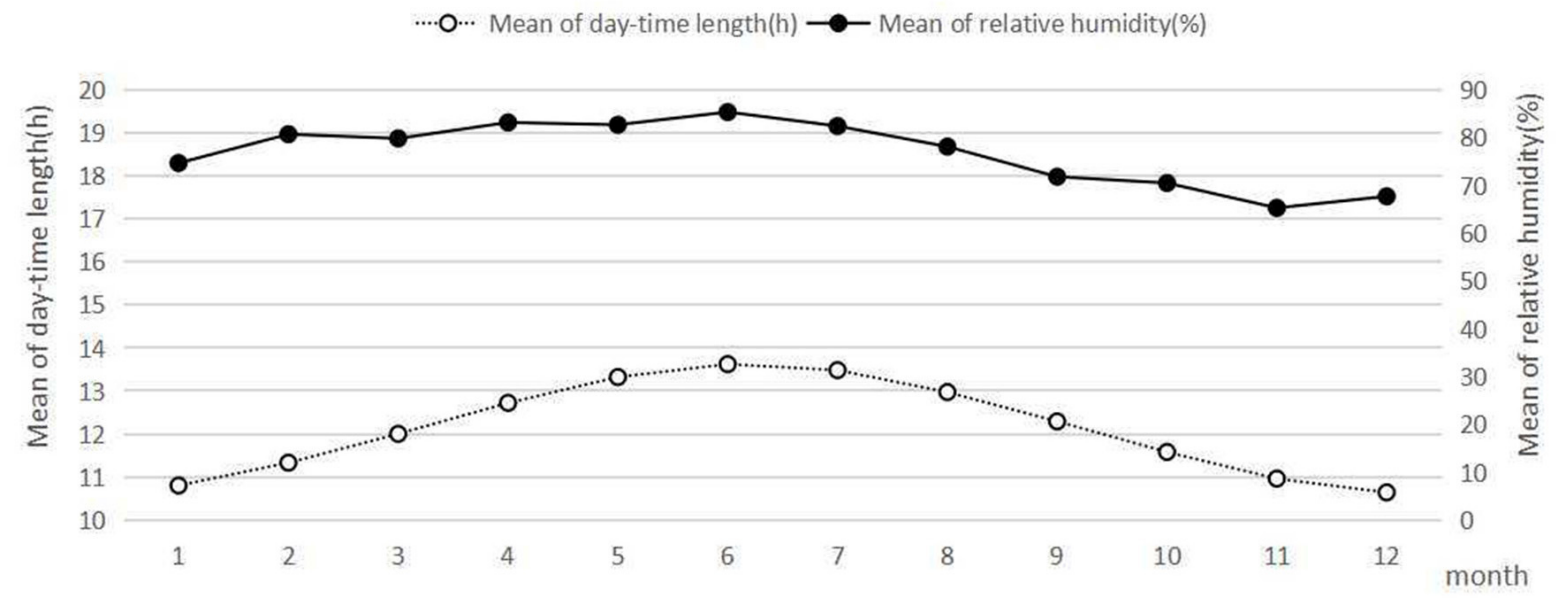
Climatic parameters of Zhangzhou in 2019.

The mean daily maximum temperature, daily minimum temperature, daily outdoor temperature, daily daytime length, and relative humidity were calculated on a monthly basis and presented in Figures 1, 2. January, February, and December were the coldest months in 2019. From June to September, the outdoor temperature was at the highest level. One-way ANOVA indicated that the mean monthly pre-dialysis SBP and DBP were significantly different (*p* < 0.05).

**Figure 3:**
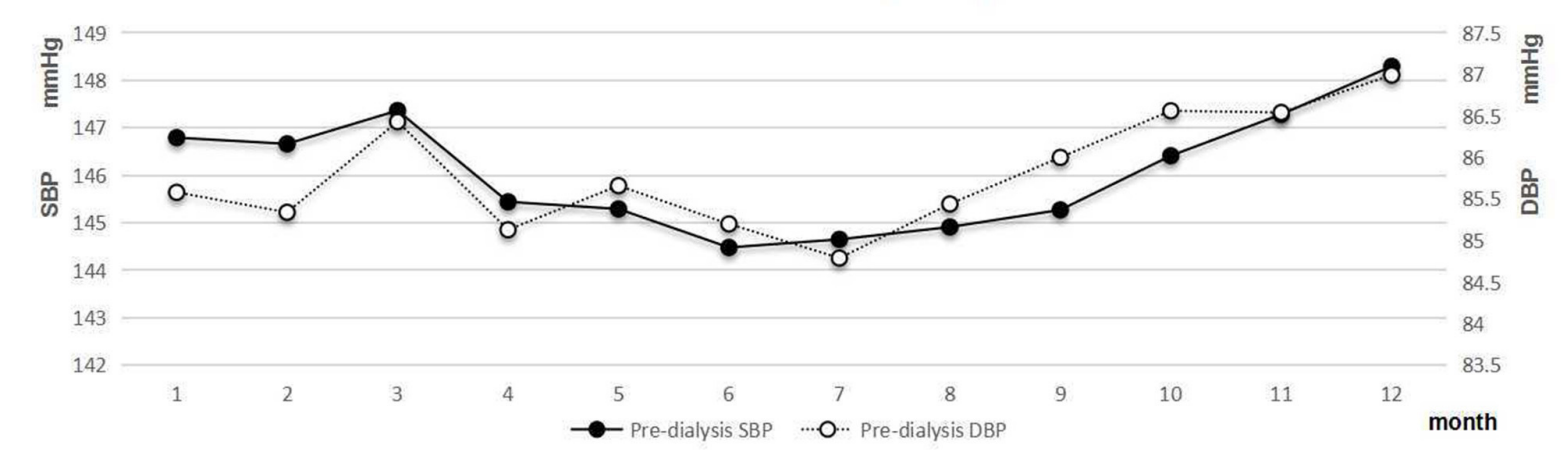
Pre-dialysis systolic and diastolic blood pressure of patients with hemodialysis.

A clear seasonal variation of pre-dialysis BP was observed (Figure 3). The change was that BP decreased with increasing temperature. The mean level of pre-SBP was the highest in December (148.29 ± 23.27 mmHg). From June to August, the mean level of pre-SBP was lower than 145 mmHg. The difference between the peak and the nadir values of mean pre-dialysis SBP was 3.81 mmHg. A similar seasonal variation of pre-dialysis DBP was also observed. The difference between the peak and the nadir values of mean pre-dialysis DBP was 2.20 mmHg.

### HD Ultrafiltration Volume

The mean value of HD UV among 38,970 HD therapies was 2,880 ± 998 ml/HD. A similar seasonal variation was observed in HD UV. In June, July, and August, the mean UV was <2,800 ml. February had the highest UV (Table 1).

### Seasonal Variations in Pre-dialysis SBP and DBP in Men and Women

**Figure 4:**
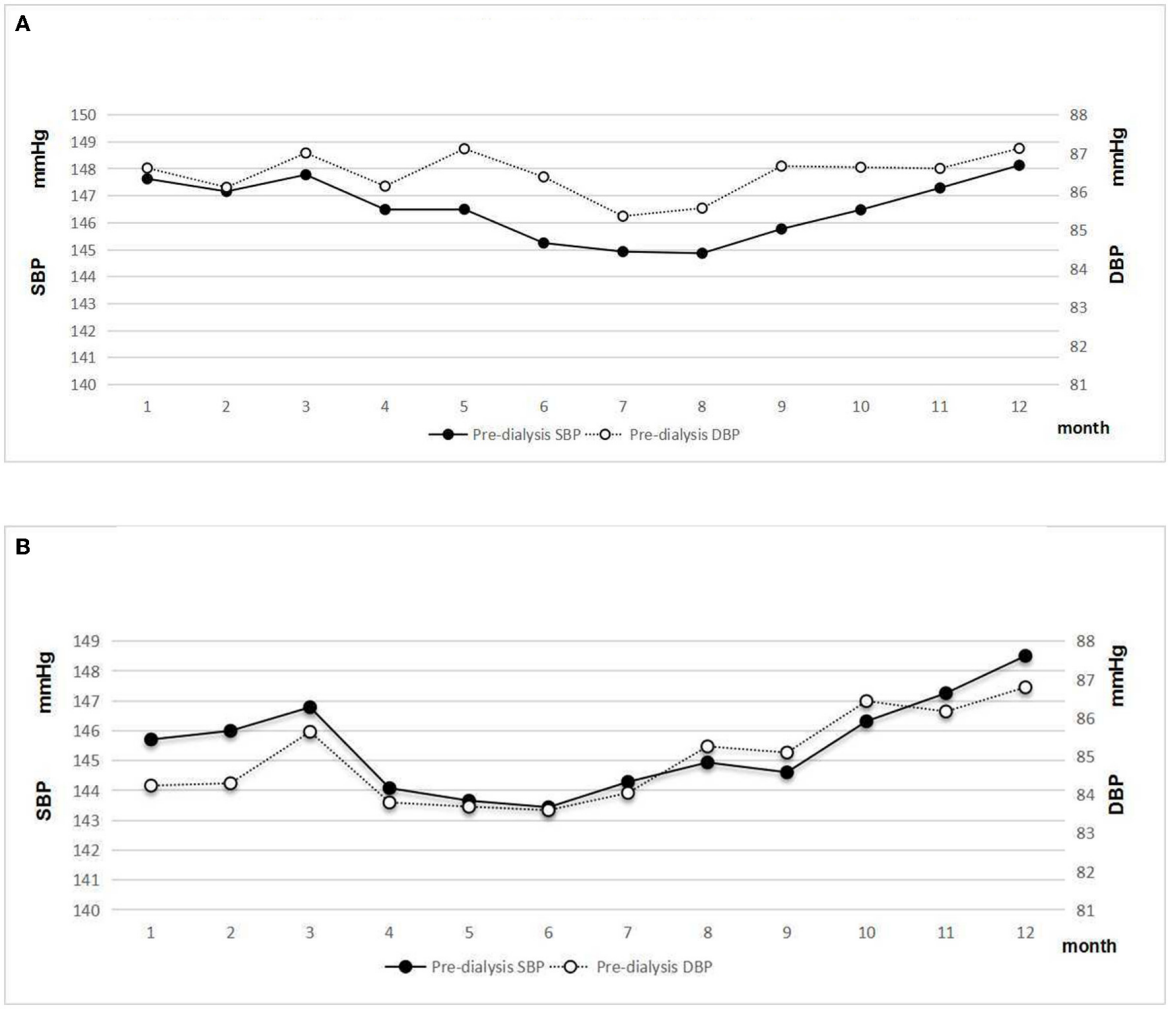
**(A)** Pre-dialysis systolic and diastolic blood pressure of male patients. **(B)** Pre-dialysis systolic and diastolic blood pressure of female patients.

Male patients with HD had a higher level of pre-dialysis SBP and DBP than female patients (*p* < 0.05). A clear seasonal variation of pre-dialysis SBP and DBP was observed in both sexes (Figures 4A,B). The greatest difference in mean monthly pre-SBP was 3.26 mmHg in men and 5.07 mmHg in women, respectively. The greatest difference in mean monthly DBP was 1.76 mmHg in men and 3.20 mmHg in women.

### Seasonal Variations in Pre-dialysis SBP and DBP in DN and Non-DN Subgroups

**Figure 5:**
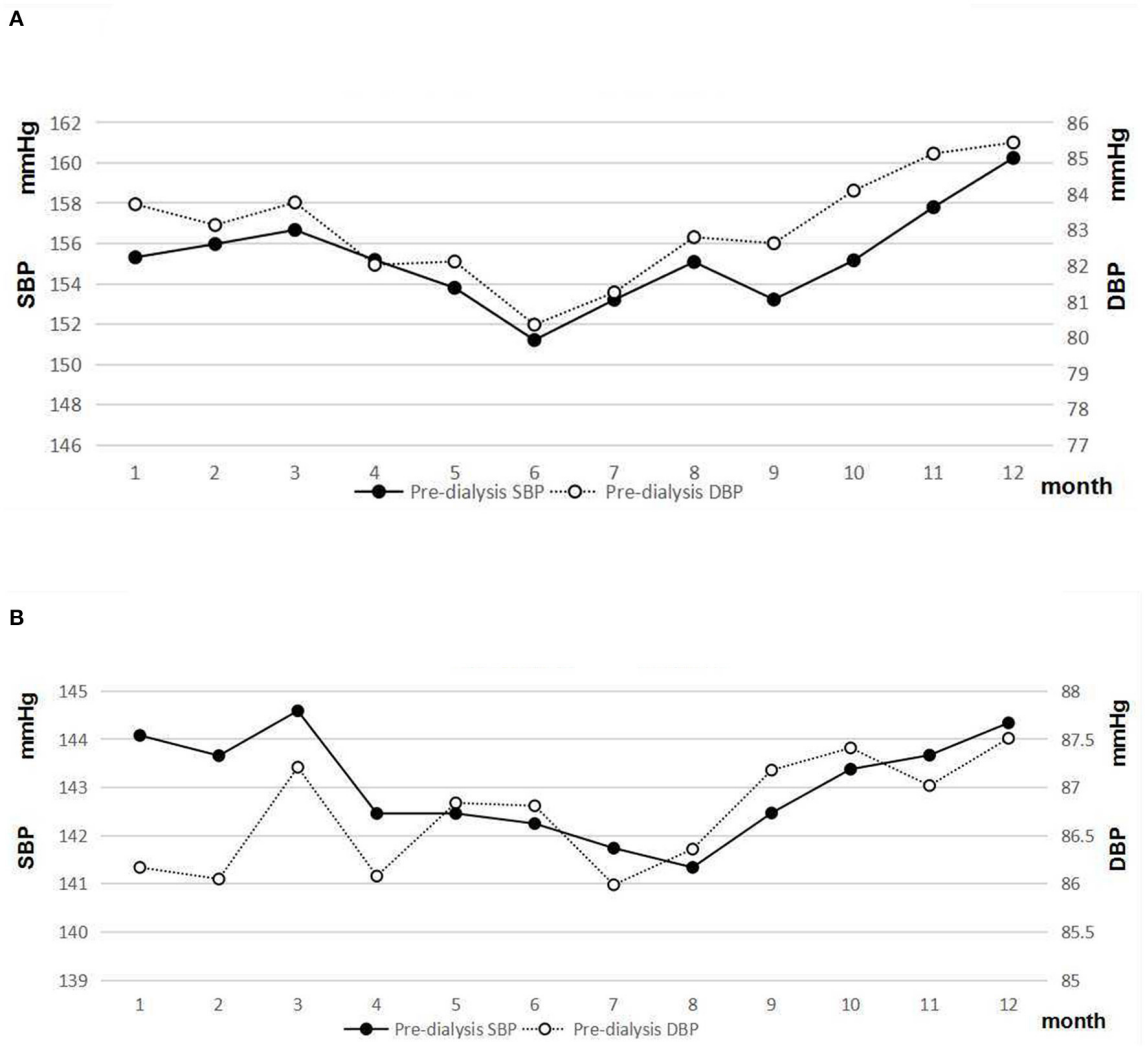
**(A)** Pre-dialysis systolic and diastolic blood pressure of patients with DN. **(B)** Pre-dialysis systolic and diastolic blood pressure of patients with non-DN.

Patients with DN had a significantly higher level of pre-dialysis SBP and a lower level of DBP than patients with non-DN (*p* < 0.05). The result indicated that the pulse pressure significantly increased in patients with DN. Although a similar seasonal variation of pre-dialysis BP was observed in both patients with DN and non-DN, the magnitude of a seasonal effect on pre-dialysis BP was greater in patients with DN (Figures 5A,B). The difference between peak and nadir values of pre-dialysis BP was 9.03/5.08 mmHg in patients with DN.

### Seasonal Variations in Pre-dialysis SBP and DBP in Different Age Subgroups

**Figure 6:**
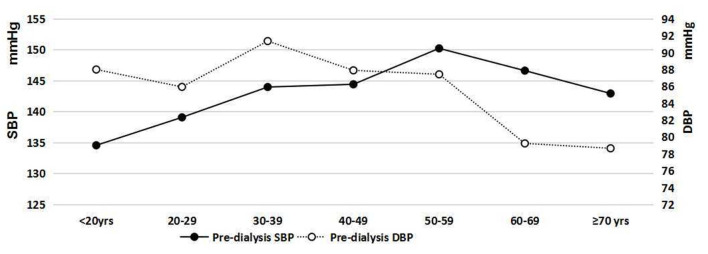
Pre-dialysis blood pressure in different age groups.

**Figure 7:**
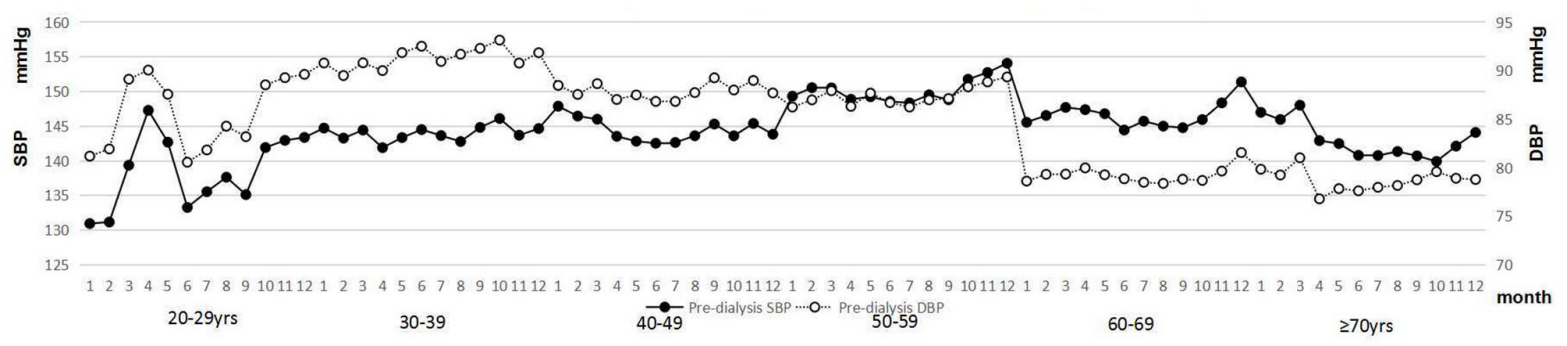
Pre-dialysis blood pressure in different ages on a monthly basis.

Pre-dialysis SBP and DBP in different age subgroups are indicated in Figure 6. Seasonal variations in pre-dialysis SBP and DBP in different age subgroups are presented in Figure 7. The results of one-way ANOVA indicated significant differences in pre-dialysis SBP in different months among different age subgroups, except for the age subgroup of 30–39-years. Significant differences in pre-dialysis DBP in different months were also found in different age subgroups, except for the age subgroup of 40–49 years and the age subgroup ≥ 70.

### Associations Between Pre-dialysis BP and Climatic Parameters

The adjusted models indicated that associations of pre-dialysis SBP and DBP with climatic parameters (the mean of daily maximum temperature, the mean of daily minimum temperature, the mean of daily temperature, the mean of daytime length, and the mean of relative humidity) were significant (*p* < 0.05) (Table 5). These associations were independent of age, gender, primary diseases, HD vintage, and UV. In the adjusted models (adjusted for age, male, vascular access, UV, primary disease, HD vintage, and the mean of daily temperature), primary disease and HD vintage were also significantly associated with pre-dialysis SBP and DBP.

**Table 5:** Association between pre-dialysis BP and climatic parameters.

	**Unadjusted model**	***P* value**	**Adjusted model**	***P* value**
**Pre-dialysis SBP**				
Mean of daily maximum temperature	−0.20(−0.24, −0.15)	<0.001	−0.15(−0.21, −0.10)	<0.001
Mean of daily minimum temperature	−0.22(−0.27, −0.17)	<0.001	−0.18(−0.23, −0.12)	<0.001
Mean of temperature	−0.21 (−0. 26, −0.17)	<0.001	−0.17 (−0. 22, −0. 12)	<0.001
Mean of daily daytime length	−0.02 (−0. 02, −0.01)	<0.001	−0.015 (−0. 019, −0. 01)	<0.001
Mean of relative humidity	−0.12 (−0. 16, −0.09)	<0.001	−0.12 (−0. 16, −0.08)	<0.001
**Pre-dialysis DBP**				
Mean of daily maximum temperature	−0. 06 (−0.09, −0.02)	0.001	−0.05(−0.05, −0.09)	0.005
Mean of daily minimum temperature	−0.07(−0.11, −0.04)	<0.001	−0.18(−0.23, −0.12)	<0.001
Mean of temperature	−0.07 (−0. 10, −0.03)	0.001	−0.17(−0. 22, −0. 12)	<0.001
Mean of daily daytime length	−0. 008 (−0. 01, −0. 005)	<0.001	−0.01 (−0. 02, −0. 01)	<0.001
Mean of relative humidity	−0. 08 (−0. 11, −0. 06)	<0.001	−0.12 (−0. 16, −0.08)	<0.001

*Adjusted for age, gender, vascular access, ultrafiltration volume, primary disease, hemodialysis vintage*.

### The Prevalence of Intradialytic Hypertension

Among 38,970 cases of HD therapy, intradialytic hypertension occurred in 23.17% of HD sessions. Older adult patients with HD had a significantly higher prevalence of intradialytic hypertension (36.45% in patients ≥ 70 years old).

## Discussion

In this retrospective study, we included nearly 40,000 pre-dialysis BP measurements in a single HD center. Zhangzhou City, located in southern China, is at the latitude near the Tropic of Cancer. The results from our study demonstrated a clear seasonal variation of pre-dialysis BP in general patients with HD, in men and women, and patients with DN and non-DN. It is consistent with the results of most previous studies based on patients with HD (9, 11, 15). A significant association of pre-dialysis SBP and DBP with climatic parameters was found in this study. The pre-dialysis BP was inversely correlated with outdoor temperature, daytime length, and relative humidity. December seemed to be a peak in the values of pre-dialysis SBP and DBP. The nadir values of pre-dialysis SBP and DBP were observed in June and July, respectively.

Since 1961, considerable studies demonstrate that seasonal variation affects BP levels (7). After 7 years of follow-up, 23,000 individuals were recruited from 10 diverse Chinese regions, concluding that mean SBP was significantly higher in the winter than in the summer (145 vs. 136 mmHg) (16). Above 5°C, every 10°C decrease in outdoor temperature was accompanied by a 6.2-mmHg increase in SBP (16). In another previous study based on over 4,00,000 health screening records in Taiwan, mean monthly values of BP were higher in the winter than in the summer for all age groups (17). Many potential mechanisms were involved in the inverse association between BP and temperature (7). The human thermoregulatory system has the function of rapid self-regulation (7, 18). Cold-induced peripheral vasoconstriction is essential for keeping the body constant temperature (18). Meanwhile, cold-induced peripheral vasoconstriction leads to an increase in peripheral resistance (18). The sympathetic nervous system plays an important role in regulating BP. The activity of the sympathetic nervous system is also responsible for outdoor temperature (7, 18). Winter is accompanied by reducing the amount of sunshine (19). The inverse association of sunlight exposure with BP has been found (19). The reduction of UV light intensity is associated with a decrease in 25 (OH) vitamin D stores and increased parathyroid hormone secretion (19, 20). The change may influence endothelial function, which responds to increasing BP (20). However, Rostand et al.'s study suggested that 25 (OH) vitamin D could not explain the association between greater sunlight exposure and the reduction of BP (19). Other factors, such as decreased sweating, changes in dietary habits, exercise, and various behavioral habits, may contribute to a seasonal variation in BP (7).

The special feature of the present study is that the subjects are patients with HD. The regulation of BP is very complex and multifactorial (21). In clinical studies of relevant BP, frequent and close BP monitoring is required. However, it is difficult to ensure the compliance of family self-monitoring BP for a long time. Therefore, a few large-scale studies on a seasonal variation of BP only took a short-term period monitoring of BP to analyze (22, 23). Patients who are undergoing HD are a special group. HD therapy plays a key role in maintaining patient survival. For this reason, patients must follow the therapeutic schedule. In clinical practice, we have the opportunity to closely monitor patients with HD. Thus, we accumulate a large amount of data of patients with HD. In this study, we analyze the data on 38,970 HD sessions. BP measurements during each HD were included in our data. Even compared with similar studies based on patients with HD (9, 11), our data are more complete and have better continuity.

An inverse association between pre-dialysis BP of outdoor temperature and relative humidity is observed in our study. Pre-dialysis BP increased in the winter by 3.81/2.20 mmHg (SBP/DBP) in comparison to the summer. The magnitude of a seasonal variation of pre-dialysis BP is relatively small in this study. The change in pre-dialysis BP from the peak to nadir value was 8/7 mmHg (9). The BP seasonal variability is related to a variation in outdoor temperature. Maximal monthly temperatures ranged from 10°C in the winter to 31°C in the summer in the ARGILéS's study (9). In Zhangzhou City, mean monthly maximal temperatures varied from 20°C in January to 34°C in August. A relatively mild change in temperature may be a possible explanation for the smaller seasonal variation in BP.

Volume overload is major pathogenesis of hypertension in patients with dialysis (2, 13). The retention of water interval between the two HD therapies is a unique factor affecting BP in patients undergoing HD (2, 24, 25). In our study, we observed that UV changed with the season (higher in the winter and lower in the summer). Increased sweat in the summer results in increased salt and water loss (7). This change might contribute to BP seasonal variability (7). A strong link between BP variations and interdialytic body weight gain has been established in a previous study (26). Our study also suggested that seasonal factors are independent factors associated with BP in patients with HD.

In this study, we also explored the association with BP of climatic parameters in different subgroups. We found that seasonal BP variations exist in both men and women. Age is another factor linked to BP seasonal variability (7). The association of temperature with BP is stronger in older adults (27). However, in this study, the association between age and a seasonal variation in BP seems to be a little paradoxical. The results indicated a significant seasonal variation of SBP among different age subgroups, except for the age subgroup of 30–39 years. A seasonal variation of DBP was also not found for all different age subgroups. Probably, the association of a season variation with BP is modified in terms of age. When an analysis is performed in different age subgroups, a small sample size is not enough to interpret.

A seasonal variation of BP has also been investigated in patients with DM (28, 29). Seasonal variations of BP in patients with type 1 diabetes and type 2 diabetes were observed (28). In Ushigome's study, the summer-winter difference in morning home BP was 14.0/6.5 mmHg (29). In the current study, we also found a seasonal variation in BP for patients with an increase in DN. A difference between the peak and nadir value of BP was 9.03/5.08 mmHg in patients with DN. In patients with non-DN, this value was only 3.25/1.52 mmHg. It has been observed that seasonal variations of endothelium-dependent flow-mediated vasodilation increased with the presence of type 2 diabetes (30). This may mediate the amplification in seasonal variations of BP in patients with DN. A seasonal variation of BP is also associated with older adult age, which attributes to impaired baroreflex control and enhanced vasoreactivity (7, 27, 29, 31). In this study, patients with DN were significantly older adults than patients with non-DN.

The primary advantage of the present study was that patients with HD were a distinct group. BP seasonal change is associated with mortality and renal outcome in patients with chronic kidney disease (32). BP seasonality varies by a different subgroup (28). To our knowledge, no previous study on BP seasonality was performed in Chinese patients with HD. The second advantage of the present study is a large and complete database. We use the information system to automatically record and store data to ensure the integrity of the data.

The present study also has obvious limitations. The first caveat is that we used pre-dialysis, post-dialysis, and intradialytic BP measurements as the target. During the HD therapy, the accuracy of BP measurements was affected by many factors, including puncture pain, tension, dialysis room environment (2). Patients may be under psychological stress. BP measurements in the HD unit are not standard office BP (2, 4, 13). Even if measured using a standardized protocol, pre-dialysis and post-dialysis may be imprecise (2). Home self-measured BP and ambulatory BP are the better prognostic predictors for the patients who are undergoing HD (2, 4, 13). Ambulatory BP monitoring, the gold standard method for BP evaluation, has a superior risk prediction for mortality (2). Home BP measurement had higher short-term reproducibility, and it improved the prediction of adverse outcomes (2). However, ambulatory BP monitoring and home BP measurements are limited by patient compliance and availability (2). Despite the abovementioned limitations, the values of peridialytic BP measurements are still of clinical importance (2). Considering that the abovementioned BP measurements were obtained under similar conditions, we believe that these BP measurements are still comparable. Second, we must point out that we used all BP measurements from one HD unit. BP measurements for every month were not obtained exactly from the same group. The main causes of change of patients included initiating HD, transferring to other HD units, receiving transplantation, and death. However, age, gender, DN among the BP measurements did not significantly change on a monthly basis (not presented in the result). Third, among 330 patients with an autologous arteriovenous fistula, 18 patients had a right autologous arteriovenous fistula. Because of the arteriovenous fistula, BP was measured on the nonfistula arm. This is also one of the factors affecting BP measurement. We also cannot avoid the disadvantages of a retrospective study.

## Conclusion

A clear seasonal variation in BP is observed for patients undergoing HD. Pre-dialysis SBP and DBP were inversely associated with outdoor temperature, daytime length, and relative humidity. Peak and nadir values of pre-dialysis SBP and DBP were observed in December and June to July, respectively. A seasonal variation of BP increases in patients with DN. A difference between peak and nadir values of BP is 9.03/5.08 mmHg in patients with DN. Seasonal factors should not be neglected in clinical practice.

## Data Availability Statement

The raw data supporting the conclusions of this article will be made available by the authors, without undue reservation.

## Ethics Statement

The present study has been approved by the Ethics Committee of Zhangzhou Affiliated Hospital of Fujian Medical University (2021LWB165). Written informed consent from the participants' legal guardian/next of kin was not required to participate in this study in accordance with the national legislation and the institutional requirements.

## Author Contributions

SC: design research, writing paper, and data analysis. ZW: data collection, processing, analysis, and writing paper. SL: data collection, processing, and analysis. CC: dialysis technician and provide technical support for data collection. YZ and XZ: involving in data collection. All authors contributed to the article and approved the submitted version.

## Conflict of Interest

The authors declare that the research was conducted in the absence of any commercial or financial relationships that could be construed as a potential conflict of interest.

## Publisher's Note

All claims expressed in this article are solely those of the authors and do not necessarily represent those of their affiliated organizations, or those of the publisher, the editors and the reviewers. Any product that may be evaluated in this article, or claim that may be made by its manufacturer, is not guaranteed or endorsed by the publisher.

## Footnotes

^1^ http://lishi.tianqi.com/zhangzhou/201902.html

^2^ https://richurimo.bmcx.com/zhangzhou_time_2019_01_richurimo/

^3^ http://www.wheata.cn/
