# Supplementary material for: Seasonal Variation: A Non-negligible Factor Associated With Blood Pressure in Patients Undergoing Hemodialysis
Source: Front Cardiovasc Med. 2022 Mar 18;9:820483. doi: 10.3389/fcvm.2022.820483 (PMC8971928; doi:10.3389/fcvm.2022.820483)
Supplement: Supplementary file 1 [file Data_Sheet_1.doc]

**Supplementary-1 Coefficient of variation of pre-dialysis blood pressure and post-dialysis blood pressure**

| Month | N | Pre-dialysis  SBP (mmHg) | CV of Pre-dialysis  SBP (%) | Pre-dialysis  DBP (mmHg) | CV of Pre-dialysis  DBP (%) |
| --- | --- | --- | --- | --- | --- |
| Jan. | 3090 | 146.79 ± 23.31 | 15.88 | 85.58 ±16.82 | 19.66 |
| Feb. | 2787 | 146.66 ± 23.53 | 16.04 | 85.34 ±16.77 | 19.65 |
| Mar. | 3075 | 147.36 ± 22.47 | 15.25 | 86.43 ± 16.85 | 19.50 |
| Apr. | 3131 | 145.44 ± 22.73 | 15.62 | 85.13 ± 16.37 | 19.23 |
| May | 3305 | 145.29 ± 21.71 | 14.94 | 85.66 ± 16.51 | 19.28 |
| Jun. | 3179 | 144.48 ± 22.45 | 15.54 | 85.20 ± 16.25 | 19.08 |
| Jul. | 3492 | 144.65 ± 23.13 | 15.99 | 84.79 ±16.12 | 19.01 |
| Aug. | 3423 | 144.91 ± 23.41 | 16.15 | 85.44 ±16.32 | 19.11 |
| Sept. | 3127 | 145.27 ± 23.04 | 15.86 | 86.00 ± 16.89 | 19.64 |
| Oct. | 3484 | 146.41 ± 23.23 | 15.87 | 86.56 ±16.84 | 19.46 |
| Nov. | 3426 | 147.28 ± 23.66 | 16.06 | 86.54 ± 16.03 | 18.53 |
| Dec. | 3451 | 148.29± 23.27 | 15.69 | 86.99 ± 16.48 | 18.94 |
| Total | 38970 | 146.07 ± 23.03 | 15.77 | 85.82 ±16.53 | 19.26 |

**Supplementary-2 Use of antihypertensive drugs**

| **Antihypertensive drugs** | **Jan** | **Feb** | **Mar** | **Apr** | **May** | **Jun** | **Jul** | **Aug** | **Sept** | **Oct** | **Nov** | **Dec** |
| --- | --- | --- | --- | --- | --- | --- | --- | --- | --- | --- | --- | --- |
|  |  |  |  |  |  |  |  |  |  |  |  |  |
| **CCB one tablet/d** | 60 | 59 | 68 | 65 | 66 | 65 | 61 | 50 | 58 | 64 | 46 | 52 |
| **CCB two tablets/d** | 121 | 117 | 120 | 119 | 117 | 108 | 116 | 111 | 114 | 129 | 133 | 149 |
| **CCB>2#/d** | 10 | 7 | 9 | 11 | 7 | 7 | 9 | 7 | 4 | 6 | 10 | 6 |
|  |  |  |  |  |  |  |  |  |  |  |  |  |
| **ARB one tablet/d** | 20 | 21 | 25 | 28 | 29 | 28 | 28 | 22 | 26 | 26 | 23 | 21 |
| **ARB two tablets/d** | 23 | 23 | 21 | 20 | 20 | 18 | 16 | 14 | 20 | 20 | 27 | 31 |
| **ARB>2#/d** | 1 | 1 | 1 | 2 | 1 | 3 | 2 | 2 | 1 | 2 | 2 | 1 |
|  |  |  |  |  |  |  |  |  |  |  |  |  |
| **ACEI one tablet/d** | 0 | 0 | 0 | 3 | 2 | 4 | 0 | 1 | 0 | 1 | 2 | 1 |
| **ACEI two tablets/d** | 2 | 2 | 2 | 4 | 5 | 4 | 2 | 2 | 2 | 1 | 1 | 1 |
| **ACEI>2#/d** | 0 | 1 | 1 | 0 | 0 | 0 | 0 | 0 | 0 | 0 | 0 | 0 |
|  |  |  |  |  |  |  |  |  |  |  |  |  |
| **Beta-blockers one tablet/d** | 40 | 39 | 39 | 36 | 41 | 38 | 44 | 38 | 47 | 43 | 40 | 46 |
| **Beta-blockers two tablets/d** | 24 | 30 | 29 | 32 | 29 | 31 | 28 | 23 | 21 | 23 | 25 | 26 |
| **Beta-blockers>2#/d** | 23 | 22 | 25 | 27 | 25 | 24 | 20 | 23 | 21 | 22 | 25 | 27 |
|  |  |  |  |  |  |  |  |  |  |  |  |  |

**Supplementary-3 Change on doses of antihypertensive drugs**

| **Antihypertensive drugs** | **Jan** | **Feb** | **Mar** | **Apr** | **May** | **Jun** | **Jul** | **Aug** | **Sept** | **Oct** | **Nov** | **Dec** |
| --- | --- | --- | --- | --- | --- | --- | --- | --- | --- | --- | --- | --- |
|  |  |  |  |  |  |  |  |  |  |  |  |  |
| **Began to decrease** | 0 | 9 | 8 | 15 | 17 | 18 | 26 | 14 | 7 | 11 | 6 | 1 |
| **Began to increase** | 3 | 2 | 3 | 6 | 10 | 7 | 8 | 9 | 10 | 13 | 6 | 11 |
